# Global Influence of Cannabis Legalization on Social Media Discourse: Mixed Methods Study

**DOI:** 10.2196/65319

**Published:** 2025-09-29

**Authors:** Consuelo Castillo-Toledo, Carolina Donat-Vargas, María Montero-Torres, Francisco J Lara-Abelenda, Fernando Mora, Melchor Alvarez-Mon, Javier Quintero, Miguel Ángel Álvarez-Mon

**Affiliations:** 1 Department of Medicine and Medical Specialities, Faculty of Medicine and Health Sciences, University of Alcala, Alcalá de Henares, Spain; 2 Department of Psychiatry and Mental Health, Hospital Universitario Infanta Leonor, Av. Gran Vía del Este 84, Madrid, 28031, Spain, 34 687073326; 3 Cardiovascular and Nutritional Epidemiology, Institute of Environmental Medicine, Karolinska Institute, Stockholm, Sweden; 4 IMDEA-Food Institute, Consejo Superior de Investigaciones Científicas, Universidad Autónoma de Madrid, Madrid, Spain; 5 Departamento Teoria de la Señal y Comunicaciones y Sistemas Telemáticos y Computación, Escuela Tecnica Superior de Ingenieria de Telecomunicación, Universidad Rey Juan Carlos, Fuenlabrada, Spain; 6 Department of Legal Medicine and Psychiatry, Complutense University, Madrid, Spain; 7 Ramon y Cajal Institute of Sanitary Research (IRYCIS), Madrid, Spain; 8 Service of Internal Medicine and Immune System Diseases-Rheumatology, University Hospital Príncipe de Asturias, (CIBEREHD), Alcalá de Henares, Spain

**Keywords:** cannabis, Twitter, social perception, infodemiology, drug use, geolocalization, drug abuse

## Abstract

**Background:**

Cannabis is the third most consumed drug worldwide, with its use linked to a high number of substance use disorders, particularly among young men. Associated mortality causes include traffic accidents and cardiovascular diseases. The global expansion of cannabis legalization has sparked debates about its impact on risk perception, with risk perception decreasing in countries with permissive laws. Social media analysis, such as on Twitter (subsequently rebranded as X), is a useful tool for studying these perceptions and their variation by geographic region.

**Objective:**

This study aims to analyze Twitter users’ perceptions of cannabis use and legalization, taking into account the geographic location of the tweets.

**Methods:**

A mixed methods approach was used to analyze cannabis-related tweets on Twitter, using keywords such as “cannabis,” “marijuana,” and “hashish.” Tweets were collected from January 1, 2018, to April 30, 2022, in English and Spanish, and only those with at least 10 retweets were included. The content analysis involved an inductive-deductive approach, resulting in the classification of tweets into thematic categories, including discussions on legalization.

**Results:**

The tweet analysis showed that in America, Europe, and Asia, political discussions about cannabis were the most common topic, while personal testimonies dominated in Oceania and Africa. In all continents, personal experiences with cannabis use were mostly positive, with Oceania recording the highest percentage (1642/2695, 60.93%). Regarding legalization, Oceania also led with the highest percentage of tweets in favor (1836/2695, 68.13%), followed by America and Africa, while support in Europe and Asia was slightly lower, with about half of the tweets in favor.

**Conclusions:**

The political debate has been the most frequently mentioned topic, reflecting the current situation in which legislative changes are being discussed in many countries. The predominance of opinions in favor of legalization, combined with the prevalence of positive experiences expressed about cannabis, suggests that the health risks associated with cannabis use are being underestimated in the public debate.

## Introduction

Cannabis is the third most consumed drug worldwide, after alcohol and tobacco. It is estimated that 219 million people used cannabis in 2021, which corresponds to 4% of the global population. In 2021, approximately 46% of countries reported that cannabis was the drug linked to the highest number of substance use disorders [1].

It is estimated that 22.1 million people worldwide had a cannabis use disorder [2], which is more common in men than in women. A study conducted in Australia, Europe, and the United States revealed that the average age at which cannabis use disorder manifests is 22 years [3]. The most frequent causes of mortality associated with cannabis use are traffic accidents, suicide, and cardiovascular and pulmonary diseases [4,5]. A study conducted in New Zealand concluded that the risks of driving under the influence of cannabis could be greater than the risks of driving under the influence of alcohol [6].

The increasing global legalization of cannabis is generating significant political and social debate. It is necessary to study the impact of legislative changes on the public’s perception of risk. Studies have shown that in countries where cannabis use has been legalized, the perception of risk has decreased [7]. According to the Survey on Drug Use in Secondary Education in Spain (ESTUDES) conducted in 2023, 12.9% of respondents stated that the legalization of cannabis would encourage them to try it [8].

In this context, analyzing social media posts is a useful tool for understanding public opinion, which can help to identify factors that influence cannabis use. Social media platforms are increasingly being used by researchers to monitor public health [9], as these platforms capture more sincere and spontaneous opinions than conventional opinion studies conducted in hospital settings [10,11]. A systematic review that analyzed the advantages and disadvantages of using Twitter in public health research concluded that it is a valuable tool for identifying social concerns and information needs on a given topic [12,13]. Previous studies have demonstrated the effectiveness of content analysis as a public health tool for analyzing and studying issues related to drugs [14-17].

In this study, we aim to understand how attitudes toward cannabis and its legalization vary by geographic region through the analysis of tweets made on Twitter. We hypothesize that in countries with more permissive cannabis use and purchase laws, the perception of risk decreases.

## Methods

### Search Strategy and Data Collection on Twitter

This mixed method analysis, both quantitative and qualitative, focused on the content of tweets related to cannabis posted on the social network Twitter. We collected all tweets using the following keywords (in English or Spanish): cannabis, marijuana, and hashish. We included those tweets that met the following inclusion criteria: (1) Public tweets, (2) tweets containing any of the aforementioned keywords in the text, (3) published between January 1, 2018, and April 30, 2022, (4) written in English or Spanish, and (5) having received at least 10 retweets. The inclusion criteria were selected to capture a broad and representative social media discussion on the topic. We decided to collect and analyze tweets over a period of more than 4 years to cover the broadest possible timeframe, making the study more representative. If a timeframe of only days or weeks is selected, conversations may be heavily influenced by specific events or legislative changes (eg, a change in cannabis regulations in a particular country). Most studies of this nature are limited to days, weeks, or months. Very few studies analyze social media posts (or other internet spaces) over multiple years. We could have selected a longer timeframe, but the economic cost would have increased significantly.

The tool we used to collect the tweets is Tweet Binder, which has been widely used in previous research and can access 100% of public tweets. In addition to the tweet text, this tool provides the number of retweets and likes for each tweet, as well as the date they were published, a link to the tweet in its context, the user description, and geolocation. The number of retweets and likes each tweet received is an indicator of the interest generated by the corresponding content among users.

### Content Analysis Process

Using the previously mentioned search criteria, we collected 69,033 tweets in Spanish and 181,217 tweets in English. Next, the remaining tweets were analyzed using a mixed inductive-deductive approach to develop a codebook to classify the tweets based on key thematic categories. A small subset of tweets (n=300) was manually classified by 2 members of the research team. After discussing discrepancies and adjusting the codebook by consensus, 2000 more tweets were analyzed. Finally, an automated and computerized classification of the remaining larger subset of tweets (n=250,250) was performed.

Tweets were classified as classifiable or nonclassifiable. A tweet was considered nonclassifiable when its content was unrelated to the objectives of this work, if the content was insufficient to contain relevant information, or if it was written in a way that made its meaning uncertain. For each classifiable tweet, the content was analyzed according to the following topics: (1) tweet theme, (2) personal experience with the drug, and (3) legalization. The classification criteria and examples of tweets are shown in Table 1.

**Table 1.** Category, definitions, and examples of classification. Usernames and personal names were removed.

Categories	Examples
Tweet theme	
Political discussion (refers to both police or social or political complaint or claim [for or against])	On July 1 2021 the New Jersey Supreme Court announced procedures for automatic vacation dismissal and expungement of certain marijuana- and hashish-related cases.
General information (refers to when talking about more scientific issues).	"Podemos" (communism) is always against progress and freedoms and against health. It calls for the legalization of cannabis, which destroys neurons and willpower. It causes memory and learning problems, dependency, anxiety, depression, lung diseases, cancer, arrhythmia, and psychosis...
Sale and advertising (cannabis is advertised)	The Assad regime has transformed into one of the world’s leading narcotics enterprises. Hashish is among its major drug exports but the most lucrative is captagon a mild stimulant pill consumed recreationally throughout the Middle East
Testimonials (regarding consumption, experience, more from the opinion of drug users, or families or friends)	Second-Hand Smoke Has Never Been Funnier'-Reporter: "Burning behind me is 8 1/2 tons of heroin opium hashish & other narcotics…(uncontrollable giggling)"-This is what happens when you stand too close to a pile of burning drugs. It's like taking a toke from a HUGE joint
Trivialization (minimization of the consequences of consumption, stigmatization, and humorous tweets)	This Lebanese town's residents rely on hashish to survive. The problem? It is all illegal
Personal experience with cannabis^a^	
Negative	The professor MUST 1st have to answer: WHAT KIND OF ENVIRONMENT PRODUCES VIOLENT YOUTHS AND FANATICALHASHISH SMOKING TERRORISTSlacking enterprise and ingenuity for growth?It takes a special breed of people to persevere with leeches...I commend all Biafransincluding Igbos.
Positive	Cannabis Cafe smoke while you eat add thc seasoning to your food take everything home (weed + food) food is actually torch10/10 recommend
Legalization	
No mention (or against)	Again ..The state of terrorism mercenaries and drugs (Bashar Assad and his gangs): The Jordanian army foiled an attempt to smuggle a huge shipment of drugs from Syria which consisted of nearly 1 million and 21 thousand pills of captagon and 35 hashish bricks.
In favor of legalization	Finally Morocco legalises it most famous export. They've been growing marijuana in the mountains for centuries and Moroccan hashish is cherished in Europe. This week parliament is debating a cannabis legalisation bill for "medicinal use" (😉)

a Personal experience with cannabis, whether through acquaintances, friends, family members, or personal use, or related to social events associated with its consumption.

### Machine Learning Classifier

The methodology followed in this project has been validated in prior research studies [18,19]. First, a preprocessing of the database should be executed. This preprocessing involves a translation of the non-English tweets to English using Google Translate (3.1.0a0 version) and a normalization of the tweets by removing special characters, splitting negative contractions, and removing repetitions. Then, we use a pretrained network called BERTWEET, trained on 850 million English tweets [20], to classify cannabis-related tweets. Since BERTWEET was not initially designed for the specific classification categories, fine-tuning was performed. Manually classified tweets were randomly divided into an 80% training subset and a 20% testing subset. The training subset was used to fine-tune the network, while the testing subset was used to validate its performance. In addition, to address some imbalanced categories (where certain options had a higher number of tweets compared with others), text augmentation was performed using the library called textattack (0.3.8 version; (QData research group) [21].

### Statistical Analysis

The results were presented in tables or figures, showing the percentage of tweets in each category. To compare the proportions of tweets between categories, Pearson chi-square test was used, yielding a *P* value indicating statistical significance.

Choropleth maps were generated as a visualization tool to depict the global distribution of tweets. In addition, these maps were used to illustrate the geographic distribution of tweets expressing support for the legislation and exhibiting a sentiment favorable to cannabis.

The statistical analyses were performed using the software packages STATA v16 (StataCorp) and Microsoft Excel.

### Ethical Considerations

This study was conducted in accordance with the ethical research principles outlined in the Declaration of Helsinki (seventh revision, 2013) and received approval from the Ethics Committee of the University of Alcalá (CEID/2023/6/118). In addition, it did not directly involve human subjects or include any interventions. Only publicly available tweets were used (subject to universal access via the internet in accordance with the Terms of Service that all users agree to on Twitter). In any case, we have taken care not to disclose any usernames directly in this work and have avoided citing information that could identify specific individuals.

## Results

Out of the total number of classifiable tweets (206,526), 68.81% (142,139/206,526) had geolocation data. The continent with the highest number of tweets is America, accounting for 54.23% (112,004/206,526) of the total, followed by Europe with 9.21% (19,024/206,526), Asia with 2.39% (4946/206,526), Africa with 1.68% (3470/206,526), and Oceania with 1.3% (2696/206,526).

**Figure 1.**
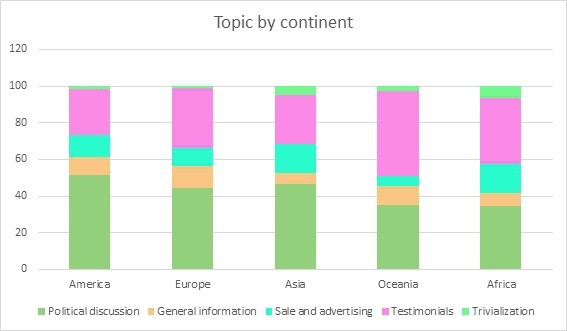
Thematic distribution by continent.

Regarding the distribution of tweets by theme, in America, Europe, and Asia, the most common theme is political discussion, while in Oceania and Africa, the most frequent theme is personal testimonies (Figure 1). The *P* value is <.001, indicating statistical significance.

**Figure 2.**
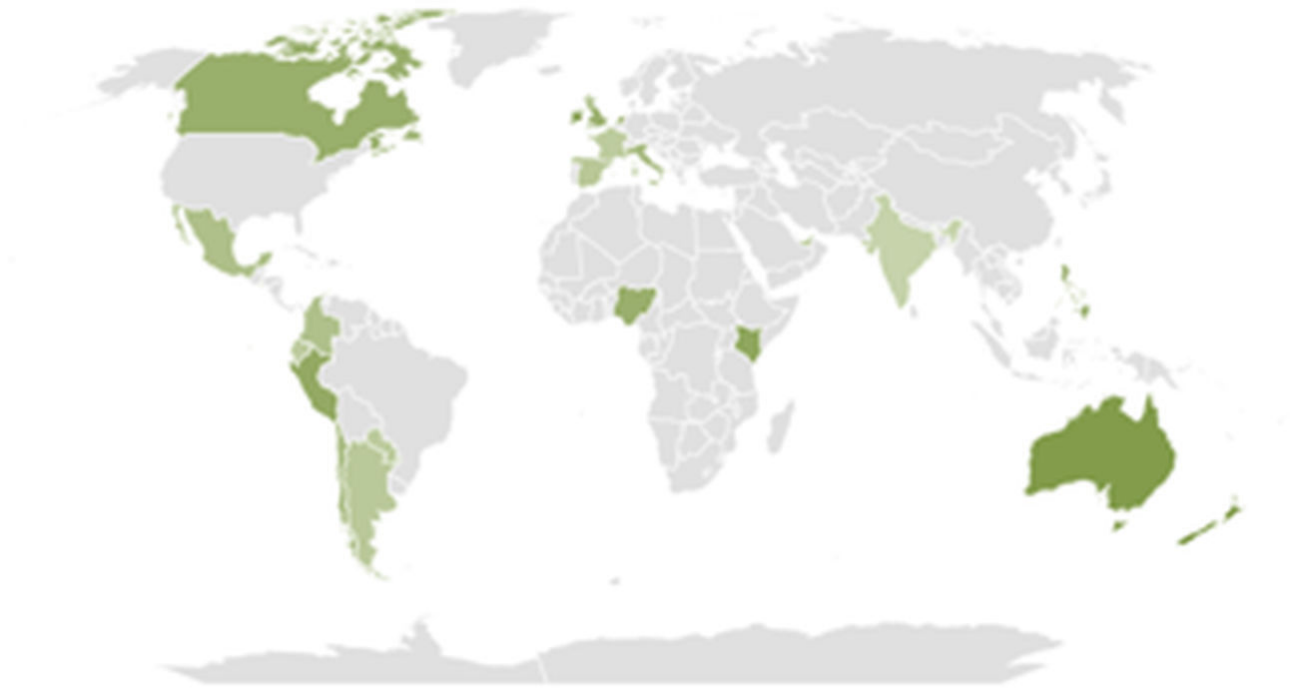
Percentage of tweets of positive experience with cannabis by continent. In dark green are the countries with the highest percentage of tweets in this category, in light green are those with fewer tweets. In gray are the geographical regions where there are no tweets about positive experiences.

Regarding personal experiences related to cannabis consumption, on all continents, there are more tweets describing positive experiences than negative ones, with Oceania having the highest percentage of such tweets. Specifically, in Oceania, we found that 60.93% (1642/2695) of the tweets described a positive experience, while only 9.09% (245/2695) described a negative experience, with 29.98% of tweets not expressing any opinion on the matter. In Africa, 50.63% (1757/3470) of tweets described a positive experience and 10.81% (375/3470) a negative one; in Europe, 40.49% (7702/19,024) described a positive experience and 10.19% (1939/19,024) a negative one; in America, 40.03% (44,837/112,004) described a positive experience and 8.16% (9144/112,004) a negative one; and in Asia, 34.07% (1685/4946) described a positive experience and 11.89% (588/4946) a negative one (Figure 2). *P* value <.001 indicates statistical significance.

**Figure 3.**
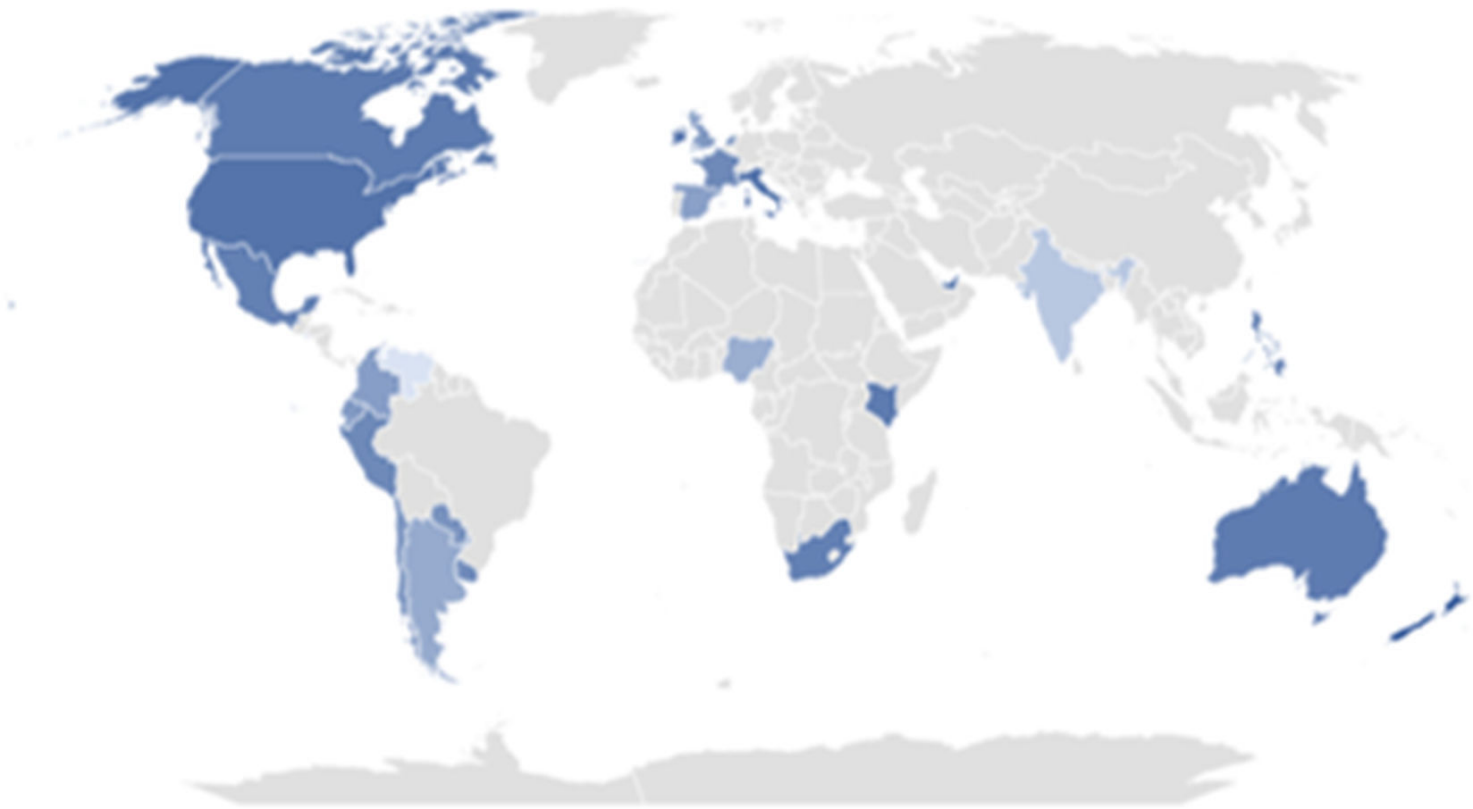
Percentage of tweets in favor of legalization. In dark blue are the countries where the most tweets in favor of cannabis legalization have been found, and as the blue gets lighter, the number of tweets decreases. In gray are the countries where there are no tweets in this category.

Finally, regarding cannabis legalization, Oceania has the highest percentage of tweets in favor of legalization, with more than double the number of tweets supporting legalization (1836/2695, 68.13%) compared to those against it (859/2695, 31.87%). In America and Africa, the percentage of tweets in favor is also higher than those expressing opposition to legalization, with 59.21% (66,313/112,004) and 53.54% (1858/3470) of tweets in favor, respectively. In Europe and Asia, the proportion of tweets supporting legalization is slightly lower, with 48.26% (9162/19,024) in Europe and 47.21% (2335/4946) in Asia (Figure 3). *P*<.001 indicates statistical significance.

## Discussion

### Principal Findings

In this analysis conducted on the platform Twitter, 68.81% (142,139/206,529) of the tweets were geolocated. The study reveals that America is the continent with the most tweets about cannabis (112,004/206,529, 54.23%), followed by Europe (19,024/206,529, 9.21%) and Asia (4,946/206,529, 2.39%). Political discussion is the most common topic in America, Europe, and Asia, while personal testimonials dominate in Oceania and Africa. Across all continents, tweets about positive experiences with cannabis outnumber negative ones, with Oceania showing the highest percentage of positive experiences (1642/2695, 60.93%). Oceania also leads in support for cannabis legalization (1836/2695, 68.13%), followed by America (66,313/112,004, 59.21%) and Africa (1858/3470, 53.54%).

Currently, more than 40 countries have enacted laws allowing cannabis use for medicinal or recreational purposes, with significant legislative variability among them. Some countries permit both recreational and medicinal use, others allow only medicinal use, some have decriminalized possession and use for profit, while others maintain it as illegal [22]. The global debate on cannabis legalization is a multifaceted issue involving social, economic, medical, and legal aspects. Proponents of legalization argue that it allows for market regulation, which can lead to a reduction in criminal activity and associated costs, as well as positive economic impacts [23]. Arguments against cannabis legalization include its potential negative impact on public health [24], effects on the brain development of young people [25], and the potential increase in cases of driving under the influence of cannabis [26]. A study conducted in the United States to evaluate the impact of legalization found that it led to decreased prices and increased availability, which may have contributed to higher cannabis consumption among adults. In addition, there was an observed increase in emergency room visits and hospitalizations related to cannabis use [27]. A systematic review indicated that recreational legalization might be associated with an increase in traffic fatalities [28]. Furthermore, states in the United States where recreational cannabis use was legalized saw rises in self-harm and assault rates [29].

Among the 5 continents, America has the highest number of countries with both recreational and medicinal cannabis legalization, with a longer historical background, particularly in countries such as the United States and Canada. This is reflected in the high volume of tweets and the prevalence of political discussions, as these countries have been at the center of debates on legalization. The greater experience and acceptance of cannabis use in these countries may explain the high proportion of tweets with positive experiences. Regarding the perception of risk related to health and cannabis consumption, legalization has been found to not only increase consumption rates but also reduce the perception of harm and decrease treatment rates for cannabis use disorders [24]. A study conducted in the United States found that in states where medicinal use is legalized, there is a higher prevalence of daily use and cannabis use disorders [30].

Europe shows a greater inclination toward political discussion, reflecting the complexities and variations in legalization among its countries. The near parity in tweets both in favor of and against legalization in Europe suggests an active and diverse debate environment, with divided and evolving opinions. Indeed, during the time period covered by this study, no country had legalized recreational use, although countries such as the Netherlands and Portugal had decriminalized it and approved medicinal use. In addition, this debate aligns with the 3 proposed laws to legalize recreational cannabis use in Spain in 2021 [31].

As in Europe, the results reflect that there is also a debate about legalization in Asia. In this continent, the only country where recreational cannabis use was legalized in 2021 was Thailand. Therefore, research in this area is limited to this country, where an increase in cannabis consumption has also been observed following the policy changes associated with recreational cannabis use [32].

Finally, the high number of tweets about positive personal experiences found is concerning, as when a greater proportion of messages highlight positive experiences, the public is more likely to perceive consumption as safe or with fewer negative consequences [33]. Legalization may be influencing this perception. In a study conducted in California after the legalization of recreational cannabis use, the policy change was associated with perceptions of health benefits and cannabis consumption [34]. Similarly, in another study where an online survey was conducted with 16,000 Americans, it was observed that states where recreational cannabis use is legalized are more likely to believe in the benefits of marijuana [35]. The legalization of cannabis is a complex issue that involves not only medical aspects but also the need to convey to the public that its legalization does not make it less harmful. The effects of cannabis on health vary based on multiple factors such as the amount consumed, frequency of use, potency of the substance, and age, with younger individuals being the most susceptible [25]. Frequent cannabis use is associated with substance use disorders, such as those involving alcohol, tobacco, or cocaine [36], and poses a higher risk of developing depression [37] and suicidal behaviors [38,39]. A meta-analysis published in *JAMA Psychiatry* demonstrates that cannabis use during adolescence is associated with an increased risk of developing depression in adulthood and a higher risk of suicide [40,41]. Finally, cannabis use, especially in high doses or among individuals with a genetic predisposition, can increase the risk of developing psychotic disorders, such as schizophrenia [42]. Other substances, such as tobacco, while legal and socially accepted, are objectively harmful to health.

### Limitations

This study has several limitations. First, the social, economic, and demographic characteristics of Twitter users do not accurately reflect the broader population. Users of this social network tend to be younger adults with higher education levels, and it is more popular in North America, Europe, and Asia. Second, the design of the coding book and the analysis of tweets involve some subjectivity, which is common in qualitative studies. However, this methodology is consistent with previous medical research studies. Third, certain colloquial terms for cannabis, such as “dope” or “maría,” had to be excluded as keywords due to their multiple meanings, potentially resulting in lost sample data and information on the topic. Fourth, the opinions of Twitter users may vary over time, just as political debate topics do, so the conclusions of this study should be interpreted with caution. Fifth, another limitation is the difficulty of conducting an analysis based on the legal status of cannabis at a global level. In some countries, recreational cannabis use was legal before data collection began, while in others, it remained illegal until the end of the study period. Likewise, in the United States, legislation varies by state, and our database only identifies that the tweet originates from the country without specifying the state of origin. These legal differences introduce nuances that limit the validity of a comparative analysis. Finally, another limitation of this study is the potential variability in sampling and the availability of geolocated data on the Twitter platform. Twitter data is not always accurate or fully geocoded, which may affect the geographical representation of the results. Although geolocated information was used for some analyses, the lack of geolocation in many tweets may have influenced the observed distribution, limiting the ability to obtain an exact picture of the origin of the tweets. In addition, the limitation of using tweets published only in English or Spanish may explain why continents such as Africa or Asia are underrepresented. Future work should consider including more widely spoken languages in these continents.

### Conclusions

The political debate has been the most frequently mentioned topic in relation to cannabis, reflecting the current situation in which legislative changes regarding cannabis are being discussed in many countries. On this social media platform, tweets in favor of legalization have predominated. Across all continents, positive experiences with cannabis have outweighed negative ones. These results suggest the need to incorporate information about the health risks associated with cannabis use, as it appears that these are being underestimated in the public debate.

## Abbreviations

ESTUDES: Survey on Drug Use in Secondary Education in Spain

## References

[1] (2021). World drug report 2021. www.unodc.org/unodc/en/data-and-analysis/wdr2021.html.

[2] (2022). Monografía cannabis 2022. consumo y consecuencias [report in Spanish]. https://pnsd.sanidad.gob.es/profesionales/publicaciones/catalogo/catalogoPNSD/publicaciones/pdf/2022_OEDA_Monografia_Cannabis.pdf.

[3] Solmi M, Radua J, Olivola M (2022). Age at onset of mental disorders worldwide: large-scale meta-analysis of 192 epidemiological studies. Mol Psychiatry.

[4] Calabria B, Degenhardt L, Hall W, Lynskey M (2010). Does cannabis use increase the risk of death? Systematic review of epidemiological evidence on adverse effects of cannabis use. Drug Alcohol Rev.

[5] Drummer OH, Gerostamoulos D, Woodford NW (2019). Cannabis as a cause of death: A review. Forensic Sci Int.

[6] Fergusson DM, Horwood LJ, Boden JM (2008). Is driving under the influence of cannabis becoming a greater risk to driver safety than drink driving? Findings from a longitudinal study. Accid Anal Prev.

[7] Brooks E, Gundersen DC, Flynn E, Brooks-Russell A, Bull S (2017). The clinical implications of legalizing marijuana: are physician and non-physician providers prepared?. Addict Behav.

[8] Observatorio español de las drogas y las adicciones (OEDA). Plan Nacional sobre Drogas.

[9] Eysenbach G (2009). Infodemiology and infoveillance: framework for an emerging set of public health informatics methods to analyze search, communication and publication behavior on the internet. J Med Internet Res.

[10] Gaspar R, Pedro C, Panagiotopoulos P, Seibt B (2016). Beyond positive or negative: qualitative sentiment analysis of social media reactions to unexpected stressful events. Comput Human Behav.

[11] Alvarez-Mon MA, de Anta L, Llavero-Valero M (2021). Areas of interest and attitudes towards the pharmacological treatment of attention deficit hyperactivity disorder: thematic and quantitative analysis using twitter. J Clin Med.

[12] Alvarez-Mon MA, Llavero-Valero M, Sánchez-Bayona R (2019). Areas of interest and stigmatic attitudes of the general public in five relevant medical conditions: thematic and quantitative analysis using twitter. J Med Internet Res.

[13] Abbasi-Perez A, Alvarez-Mon MA, Donat-Vargas C (2021). Analysis of tweets containing information related to rheumatological diseases on twitter. Int J Environ Res Public Health.

[14] Meng HW, Kath S, Li D, Nguyen QC (2017). National substance use patterns on twitter. PLoS ONE.

[15] Tofighi B, Aphinyanaphongs Y, Marini C (2020). Detecting illicit opioid content on twitter. Drug Alcohol Rev.

[16] Castillo-Toledo C, Fraile-Martínez O, Donat-Vargas C (2024). Insights from the twittersphere: a cross-sectional study of public perceptions, usage patterns, and geographical differences of tweets discussing cocaine. Front Psychiatry.

[17] Castillo-Toledo C, Fernandez-Lazaro CI, Lara-Abelenda FJ (2024). Regional insights on tobacco-related tweets: unveiling user opinions and usage patterns. Front Public Health.

[18] Butt S, Sharma S, Sharma R, Sidorov G, Gelbukh A (2022). What goes on inside rumour and non-rumour tweets and their reactions: a psycholinguistic analyses. Comput Human Behav.

[19] de Anta L, Alvarez-Mon MA, Donat-Vargas C (2023). Assessment of beliefs and attitudes about electroconvulsive therapy posted on twitter: an observational study. Eur Psychiatr.

[20] Nguyen DQ, Vu T, Tuan Nguyen A (2020). BERTweet: a pre-trained language model for english tweets. arXiv.

[21] Morris J, Lifland E, Yoo JY, Grigsby J, Jin D, Qi Y TextAttack: a framework for adversarial attacks, data augmentation, and adversarial training in NLP. https://www.aclweb.org/anthology/2020.emnlp-demos.

[22] (2024). Infografía: ¿en dónde es legal la marihuana? [Web page in Spanish]. Statista.

[23] Isorna M, Pascual F, Aso E, Arias F (2022). Impacto de la legalización del consumo recreativo del cannabis [Article in English, Spanish]. Adicciones.

[24] Assanangkornchai S, Kalayasiri R, Ratta-Apha W, Tanaree A (2023). Effects of cannabis legalization on the use of cannabis and other substances. Curr Opin Psychiatry.

[25] Scott JC, Slomiak ST, Jones JD, Rosen AFG, Moore TM, Gur RC (2018). Association of cannabis with cognitive functioning in adolescents and young adults: a systematic review and meta-analysis. JAMA Psychiatry.

[26] Asbridge M, Hayden JA, Cartwright JL (2012). Acute cannabis consumption and motor vehicle collision risk: systematic review of observational studies and meta-analysis. BMJ.

[27] Hall W, Lynskey M (2020). Assessing the public health impacts of legalizing recreational cannabis use: the US experience. World Psychiatry.

[28] Athanassiou M, Dumais A, Zouaoui I, Potvin S (2022). The clouded debate: a systematic review of comparative longitudinal studies examining the impact of recreational cannabis legalization on key public health outcomes. Front Psychiatry.

[29] Matthay EC, Kiang MV, Elser H, Schmidt L, Humphreys K (2021). Evaluation of state cannabis laws and rates of self-harm and assault. JAMA Netw Open.

[30] McBain RK, Wong EC, Breslau J (2020). State medical marijuana laws, cannabis use and cannabis use disorder among adults with elevated psychological distress. Drug Alcohol Depend.

[31] (2021). Ley cannabis: la primera propuesta de regulación de la marihuana cae en el congreso [Web page in Spanish]. La Vanguardia.

[32] Kalayasiri R, Boonthae S (2023). Trends of cannabis use and related harms before and after legalization for recreational purpose in a developing country in Asia. BMC Public Health.

[33] Rey-Brandariz J, Teijeiro A, Pérez-Ríos M (2024). Percepción del consumo de cannabis en población adolescente: metasíntesis de estudios cualitativos [Article in Spanish]. Gac Sanit.

[34] Gali K, Winter SJ, Ahuja NJ, Frank E, Prochaska JJ (2021). Changes in cannabis use, exposure, and health perceptions following legalization of adult recreational cannabis use in California: a prospective observational study. Subst Abuse Treat Prev Policy.

[35] Steigerwald S, Cohen BE, Vali M, Hasin D, Cerda M, Keyhani S (2020). Differences in opinions about marijuana use and prevalence of use by state legalization status. J Addict Med.

[36] Lynskey MT, Heath AC, Bucholz KK (2003). Escalation of drug use in early-onset cannabis users vs co-twin controls. JAMA.

[37] Martins SS, Gorelick DA (2011). Conditional substance abuse and dependence by diagnosis of mood or anxiety disorder or schizophrenia in the U.S. population. Drug Alcohol Depend.

[38] Fresán A, Dionisio-García DM, González-Castro TB (2022). Cannabis smoking increases the risk of suicide ideation and suicide attempt in young individuals of 11-21 years: a systematic review and meta-analysis. J Psychiatr Res.

[39] Shamabadi A, Ahmadzade A, Pirahesh K, Hasanzadeh A, Asadigandomani H (2023). Suicidality risk after using cannabis and cannabinoids: an umbrella review. Dialogues Clin Neurosci.

[40] Gobbi G, Atkin T, Zytynski T (2019). Association of cannabis use in adolescence and risk of depression, anxiety, and suicidality in young adulthood: a systematic review and meta-analysis. JAMA Psychiatry.

[41] Devin J, Lyons S, Murphy L, O’Sullivan M, Lynn E (2023). Factors associated with suicide in people who use drugs: a scoping review. BMC Psychiatry.

[42] Moore THM, Zammit S, Lingford-Hughes A (2007). Cannabis use and risk of psychotic or affective mental health outcomes: a systematic review. Lancet.

